# The Asian fish tapeworm *Schyzocotyle acheilognathi* is widespread in baitfish retail stores in Michigan, USA

**DOI:** 10.1186/s13071-017-2541-6

**Published:** 2017-12-22

**Authors:** Traimat Boonthai, Seth J. Herbst, Gary E. Whelan, Michelle Gunn Van Deuren, Thomas P. Loch, Mohamed Faisal

**Affiliations:** 1 Department of Pathobiology and Diagnostic Investigation, College of Veterinary Medicine, Michigan State University, East Lansing, MI 48824 USA; 2 Michigan Department of Natural Resources, Fisheries Division, Lansing, MI 48933 USA; 3 Department of Fisheries and Wildlife, College of Agriculture and Natural Resources, Michigan State University, East Lansing, MI 48824 USA

**Keywords:** Baitfish, Vector, Asian fish tapeworm, Cyprinids, Great Lakes

## Abstract

**Background:**

The Asian fish tapeworm *Schyzocotyle acheilognathi* (Yamaguti, 1934) is an important fish pathogen because of its wide range of intermediate and definitive hosts and its pathological consequences. This study was designed to determine if baitfish are a likely vector contributing to the expansion of the invasive Asian fish tapeworm.

**Results:**

We collected live baitfish for examination from 78 retail stores in Michigan between September 2015 and June 2016. A total of 5400 baitfish (90 lots, 60 fish/lot) were examined, including 42 emerald shiners [*Notropis atherinoides* (Rafinesque, 1818)] lots, 30 fathead minnow [*Pimephales promelas* (Rafinesque, 1820)] lots, 11 golden shiners [*Notemigonus crysoleucas* (Mitchill, 1814)] lots, 3 sand shiners [*Notropis stramineus* (Cope, 1865)] lots, 1 lot each of spottail shiners [*Notropis hudsonius* (Clinton, 1824)], Northern redbelly dace [*Phoxinus eos* (Cope, 1861)], and blacknose dace [*Rhinichthys atratulus* (Hermann, 1804)] and 1 lot of mixed two species: weed shiners [*Notropis texanus* (Girard, 1856)] and sand shiners.

**Conclusions:**

Based on its scolex and strobilar morphology combined with gene sequence analysis, *S. acheilognathi* was only found in emerald shiners, golden shiners and sand shiners. The mean within lot prevalence and abundance of infection was highest in emerald shiners (20.3 ± 14.0 and 1.15 ± 1.34), followed by golden shiners (8.3 ± 10.7 and 0.89 ± 1.27) and sand shiners (1.3 ± 2.6 and 0.02 ± 0.05). However, the mean intensity of *S. acheilognathi* in emerald shiners was lower (4.3 ± 2.6) than that of golden shiners (6.6 ± 6.7). *S. acheilognathi*-infected fish exhibited enlargement of the abdomen, distension of the intestinal wall, and intestinal occlusion and hemorrhage. This finding suggests that live baitfish are a likely vector by which the invasive Asian tapeworm’s range is expanding.

## Background

Aquatic invasive species and pathogens have plagued the Laurentian Great Lakes region for decades [1] and new introductions and further spread of established non-native organisms continues to be a significant threat to native communities. Detrimental aquatic organisms and pathogens have historically been introduced through numerous pathways including commercial shipping, dispersal, live baitfish trade, and stocking [2]. The risk associated with the various pathways is dynamic and gaining an understanding of the potential for invasive species and pathogens to be introduced into the landscape is critical for informed management. The live baitfish trade, for example, has been highlighted as high risk for introducing aquatic invasive species [3, 4]. The live baitfish trade pathway has limited documented evidence related to the potential to spread or introduce harmful invasive pathogens and parasites with viral hemorrhagic septicemia virus (VHSv) being a prime exception [5, 6], and the need to inspect this pathway for fish health threats is critical. Gaining information on these threats would allow managers to mitigate potential risks to reduce future ecosystem challenges resulting from new introductions.

In the Great Lakes region, the spread of the invasive Asian fish tapeworm, *Schyzocotyle acheilognathi* (Yamaguti, 1934) Brabec, Waeschenbach, Scholz, Littlewood & Kuchta, 2015 (Cestoda: Bothriocephalidae) (formerly *Bothriocephalus acheilognathi* [7]) is a great concern among managers. *Schyzocotyle acheilognathi* is a generalist invasive fish parasite that can cause substantial mortality in infected fish. Fish serve as the final host for *S. acheilognathi*, where it reaches sexual maturity in the intestinal tract and can cause significant damage in the form of intestinal occlusion, pressure necrosis, and even intestinal perforation and rupture (reviewed in [8]). Controlling the dissemination of this parasite is complicated by its two-host life-cycle between fish and cyclopoid copepods. Over 200 fish species of different families are known to serve as a definitive host [8] and the intermediate host role is played by various cyclopoid species that have very wide geographical ranges [9]. *Schyzocotyle acheilognathi* is indigenous to East Asia and was reported in the USA for the first time in 1975 [10]. This parasite has since spread to multiple states and watersheds [11, 12] where it threatens some endangered and threatened wild fish species [13, 14], as well as farmed fish [15].

Cyprinid fish species are particularly susceptible to *S. acheilognathi* parasitism, and there are increasing concerns that small freshwater cyprinids are contributing to the rapid expansion of *S. acheilognathi* in North America due to their extensive use in the baitfish trade. In this context, studies of Muzzall et al. [12] and Macrogliese et al. [16] demonstrated that several of the Great Lakes wild cyprinid species commonly used as baitfish harbor *S. acheilognathi*, which led the authors to conclude that the live baitfish trade may constitute a key pathway by which the parasite can increase its geographical range not only in the Great Lakes, but also to inland lakes, other waterways, and potentially to aquaculture facilities. In samples collected from six baitfish retail stores in Canada, Macrogliese et al. [16] detected *S. acheilognathi*, illustrating the broad spatial scope of this harmful non-native parasite.

In Michigan, the baitfish industry supports a multi-billion dollar sport fishery [17] and contributes over $20 million annually to Michigan’s economy (Michigan DNR, unpublished data). The business approach of this industry entails moving minnows from source waters to holding facilities and then to retailers using a variety of supply chains. The supply networks provide a potential vector to move invasive pathogens quickly to widely distributed waters. The majority of baitfish sold in Michigan are harvested from wild sources within the state. Specifically, the in-state catch is primarily composed of emerald [*Notropis atherinoides* (Rafinesque, 1818)] and spottail [*Notropis hudsonius* (Clinton, 1824)] shiners with lesser numbers of white suckers [*Catostomus commersonii* (Lacépède, 1803)] and sand shiners [*Notropis stramineus* (Cope, 1865)]. Capture locations for these wild minnows include shoreline locations in Saginaw Bay (Lake Huron), along Lake Huron, and the St. Clair River. In-state minnows are collected in November/December to support ice-fishing activities and again in April/May to support yellow perch [*Perca flavescens* (Mitchill, 1814)] fisheries in the spring. A lesser amount of emerald shiners and white suckers are also imported from wild sources originating from the Wisconsin River in Wisconsin. In addition, minnows in retail baitfish shops during the period from May to November are imported from other states, mainly from aquaculture operations in Arkansas, Minnesota, South Dakota and North Dakota, along with lesser amounts from wild harvest from the Wisconsin River in Wisconsin. Most of Michigan’s imported minnows from aquaculture facilities are fathead minnows [*Pimephales promelas* (Rafinesque, 1820)], golden shiners [*Notemigonus crysoleucas* (Mitchill, 1814)], and white suckers along with a smaller number of wild-caught emerald shiners and white suckers originating from waters in Wisconsin.

In general, most of the wild-caught minnows from Michigan waters are sold in the Lower Peninsula. Wild-caught emerald shiners and white suckers, mostly from Wisconsin waters, are generally sold in the Upper Peninsula. Currently, Michigan regulations prohibit the export of any wild-caught bait. Imported minnows from aquaculture facilities can be found nearly anywhere in the state. All in-state emerald shiners, spottail shiners and white suckers are inspected for VHSv and all imported minnows are inspected for VHSv and *Heterosporis* sp. While fish health inspections are conducted for some pathogens and parasites for specific baitfish species, currently there are no inspections to determine the presence of the non-native *S. acheilognathi* in the baitfish trade. Therefore, a need to determine the prevalence of *S. acheilognathi* exists and needs to be addressed to help determine if a significant risk of spread through the baitfish trade exists in Michigan.

The purpose of this study was to determine the extent to which the Asian fish tapeworm, *S. acheilognathi*, is found in the baitfish supply chain in Michigan. Given the potential damage this parasite could cause from the indiscriminate movement across the state from baitfish, this information will be used to develop new management and range expansion prevention tools.

## Methods

### Fish and sample collection

**Fig. 1:**
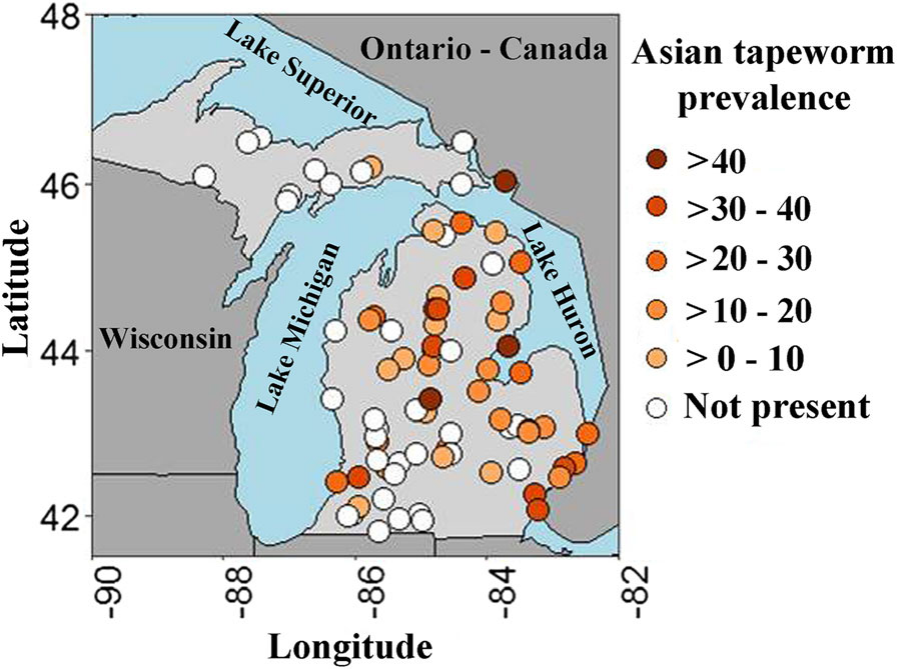
Map showing the geographical locations of sampling sites and prevalence of the Asian fish tapeworm *Schyzocotyle acheilognathi* prevalence at each location sampled

One to two lots of baitfish, each lot constituting 60 fish of single species, were anonymously purchased from retail stores (*n* = 78) throughout Michigan by the Michigan Department of Natural Resources personnel (Fig. 1). The retail stores sampled were randomly selected from a list of all licensed baitfish shops in Michigan. In addition, the baitfish lots purchased during each sampling event were later categorized as originating from an in-state or out-of-state collection source based on *post-hoc* personal communications with individual baitfish shop owners. The source categories were used to determine the effect that importing or exporting baitfish would have on the introduction or spread potential of *S. acheilognathi*. The majority (76/78) of baitfish stores kept their tanks indoors, while two stores have their tanks outdoors at the time of sample collection.

During the course of this study from September 2015 to June 2016, a total of 5400 baitfish (90 lots total) of the family Cyprinidae were examined, including 42 emerald shiner lots, 30 fathead minnow lots, 11 golden shiner lots, 3 sand shiner lots, and 1 lot each of spottail shiner, northern redbelly dace [*Phoxinus eos* (Cope, 1861)], and blacknose dace [*Rhinichthys atratulus* (Hermann, 1804)]. In one case, 60 fish of a single species were not available, and thus were replaced by 30 weed shiners [*Notropis texanus* (Girard, 1856)] and 30 sand shiners. Fish species were identified based on their original description and nomenclature was updated using FishBase (http://www.fishbase.org).

After purchase, baitfish were transported live to the Michigan State University, Aquatic Animal Health Laboratory (MSU-AAHL), where they were examined for the presence of abnormal behavioral and clinical disease signs within 24 h of collection. Next, fish were euthanized with 250 mg/l of sodium bicarbonate-buffered tricaine methane sulfonate (MS-222, Argent Chemical Laboratories, Ferndale, Washington, USA), dissected using separate sterile scissors and forceps for each fish, and examined for the presence of internal gross lesions. Last, the entire gastrointestinal tract from each fish was examined for the presence of cestodes according to USFWS and AFS-FHS [18] protocol. Gastrointestinal tracts of small fish were placed directly on a microscope slide, visceral organs removed, and then covered with another glass slide. For larger fish, contents of the gastrointestinal tracts were gently removed to keep cestode scolices intact and then spread and flattened between two microscope slides. All slides were then examined under a dissecting microscope and/or a light microscope.

### Parasitological examination and morphological identification of *S. acheilognathi*

Tapeworms with pyramidal-shaped scolices were further distinguished based on their anatomical features [10, 11, 19]. Mature cestodes were morphologically identified as *S. acheilognathi* if they displayed: (i) a pyramidal-shaped, flattened scolex that lacked hooks and suckers but had two elongated bothria; (ii) a segmented strobila; (iii) no distinct neck (i.e. the non-segmented region behind the scolex area and anterior to the first obvious segment); and (iv) a posterior portion of the scolex that was clearly wider than the anterior segmented strobila. Cestodes matching these morphological criteria were then placed in 70% ethanol for subsequent confirmatory molecular identification and enumeration.

### Molecular identification

Representative specimens that: (i) matched the morphological characteristics of *S. acheilognathi*; (ii) were recovered from multiple host species (e.g. emerald shiner, golden shiner and sand shiner); and (iii) were collected from baitfish vendors in different counties and watersheds were selected for further molecular identification. A piece of strobila (mature) or the entire tapeworm (immature) was soaked in TE buffer for 2 h on a shaking platform (200× *rpm*) to remove ethanol residue. Total genomic DNA was extracted using a DNeasy® Blood & Tissue Kit (Qiagen, Ref. no. 69506, Hilden, Germany) following the manufacturer’s protocol and then stored at -20 °C. Extracted DNA was quantified using a Qubit fluorometer (Invitrogen, Eugene, Oregon, USA) and then diluted to a concentration of 30 ng/μl for polymerase chain reaction (PCR) analysis. The near-complete internal transcribed spacer region (ITS1-5.8S-ITS2; approximate size of 1.4 kb) was PCR-amplified using the primers BD1 (5′-GTC GTA ACA AGG TTT CCG TA-3′) and BD2 (5′-TAT GCT TAA RTT CAG CGG GT-3′) [20, 21]. Each 25 μl PCR reaction was comprised of 12.5 μl of 2× GoTaq Green Master Mix (Promega, Madison, Wisconsin, USA), 0.8 μM of each primer, nuclease-free water, and approximately 30–60 ng of DNA template. PCR amplification was performed in a GeneAmp® PCR System 9700 (AB Applied BioSystems, Singapore) using a single denaturation step at 94 °C for 5 min, followed by 35 cycles of 94 °C for 30 s, 56 °C for 30 s and 72 °C for 45 s, with a final extension period of 7 min at 72 °C. Amplicons were checked visually by electrophoresis in 1.5% agarose gel containing SYBR safe (Invitrogen) under UV transillumination (UVP, Model TFM-26, Upland, California, USA). Next, amplicons were cloned into chemically competent *E. coli* (TOPO TA Cloning kit, Invitrogen, Catalog no.: K4575 J10, Carlsbad, California, USA) according to the manufacturer’s instructions and then transformants were used for further PCR-amplification using the M13 forward (5′-GTA AAA CGA CGG CCA G-3′) and M13 reverse (5′-CAG GA AAC AGC TAT GAC-3′) primers. Amplicons were again visually checked by electrophoresis, purified with the Wizard® SV Gel and PCR Clean-Up System (Promega, Ref. no. A9281, Madison, Wisconsin, USA), and then sequenced bi-directionally at the Michigan State University, Research Technology Support Facility. Sequencing chromatograms were assembled and edited using BioEdit version 7.1.3.0 [22]. Using the BLAST program [23], sequence data were compared to other cestode sequences, as well as to *S. acheilognathi* sequences from the studies of Brabec et al. [7] and Luo et al. [21] deposited in the National Center for Biotechnology Information (NCBI) database. The sequences of *S. acheilognathi* generated in this study were deposited in GenBank under the following accession numbers: KY711155–KY711166.

### Phylogenetic analysis

Sequence data from this study were aligned with one another and *S. acheilognathi* reference sequences using ClustalW in Molecular Evolutionary Genetics Analysis software (MEGA, version 6.0) [24] and then model selection for phylogenetic reconstruction was performed in MEGA 6.0, whereby the model with the lowest Bayesian information criterion (Tamura-Nei model with gamma distribution) was selected. Neighbor-joining analysis [25] was then conducted (MEGA 6.0) using the complete deletion option (total of 1124 informative sites) and topology robustness was assessed via bootstrap analysis (*n* = 1000 re-samplings).

### Statistical analyses

In this study, we used the definitions of Bush et al. [26] for prevalence (number of infected fish of one species divided by the total number of fish examined of the same species), abundance (total number of tapeworms in a fish species divided by the total number of fish examined of the same species), and mean intensity (the average number of tapeworms in a single fish host).

We used an analysis of variance (ANOVA) to determine if response variables differed among baitfish species collected throughout Michigan’s baitfish shops. Response variables included prevalence, abundance, and intensity of *S. acheilognathi*. We used the same statistical analysis to determine if response variables differed by the source or origin of where the baitfish were collected. For this analysis we had two predictor variables, which included in-state and out-of-state sources. When significant differences were detected we then used a *post-hoc* Tukey’s highly significant difference (HSD) test [27] to determine pairwise differences in mean abundance, prevalence, and intensity among the various baitfish species collected.

## Results

Baitfish were obtained between September 2015 and June 2016 from 78 randomly selected baitfish retail vendors throughout the State of Michigan (67 shops in Lower Peninsula and 11 shops in Upper Peninsula; total of 5400 baitfish; Fig. 1). Based on morphological identification combined with gene sequence analysis, *S. acheilognathi* was identified in emerald, golden and sand shiners, but not in any fathead minnow, spottail shiner, northern redbelly dace, blacknose dace, or weed shiner lots that were examined in this study (Table 1).

**Table 1:** Summary statistics of Asian fish tapeworm *Schyzocotyle acheilognathi* in fish collected from Michigan baitfish retailers

Fish species	Fish TL range (cm)	Fish weight range (g)	No. of lots examined	No. of lots infected	No. of fish examined	No. of fish infected	Mean within lot prevalence ± SD (%)	Mean within lot abundance ± SD	Mean within lot intensity ± SD
Emerald shiners	3.5–11.5	0.1–10.5	42	40	2520	512	20.3 ± 14.0	1.15 ± 1.34	4.3 ± 2.6
Golden shiners	7.8–14.5	3.3–28.4	11	7	660	55	8.3 ± 10.7	0.89 ± 1.27	6.6 ± 6.7
Sand shiners	5.0–6.5	0.7–1.4	3	1	180	3	1.3 ± 2.6	0.02 ± 0.05	0.3 ± 0.7
Fathead minnows^a^	3.5–7.2	0.4–3.9	30	0	1800	0	0	0	0
Spottail shiners	2.1–6.1	0.1–1.4	1	0	60	0	0	0	0
Northern redbelly dace	5.9–8.2	1.7–5.9	1	0	60	0	0	0	0
Blacknose dace	9.6–12.6	6.9–20.2	1	0	60	0	0	0	0
Mixed weed and sand shiners	6.8–8.4	2.3–4.6	1	0	60	0	0	0	0
Total			90	48	5400	574			

*Abbreviations: SD* standard deviation, *TL* fork length

^a^One lot was rosy fathead minnow phenotype

### Asian fish tapeworm identification

**Fig. 2:**
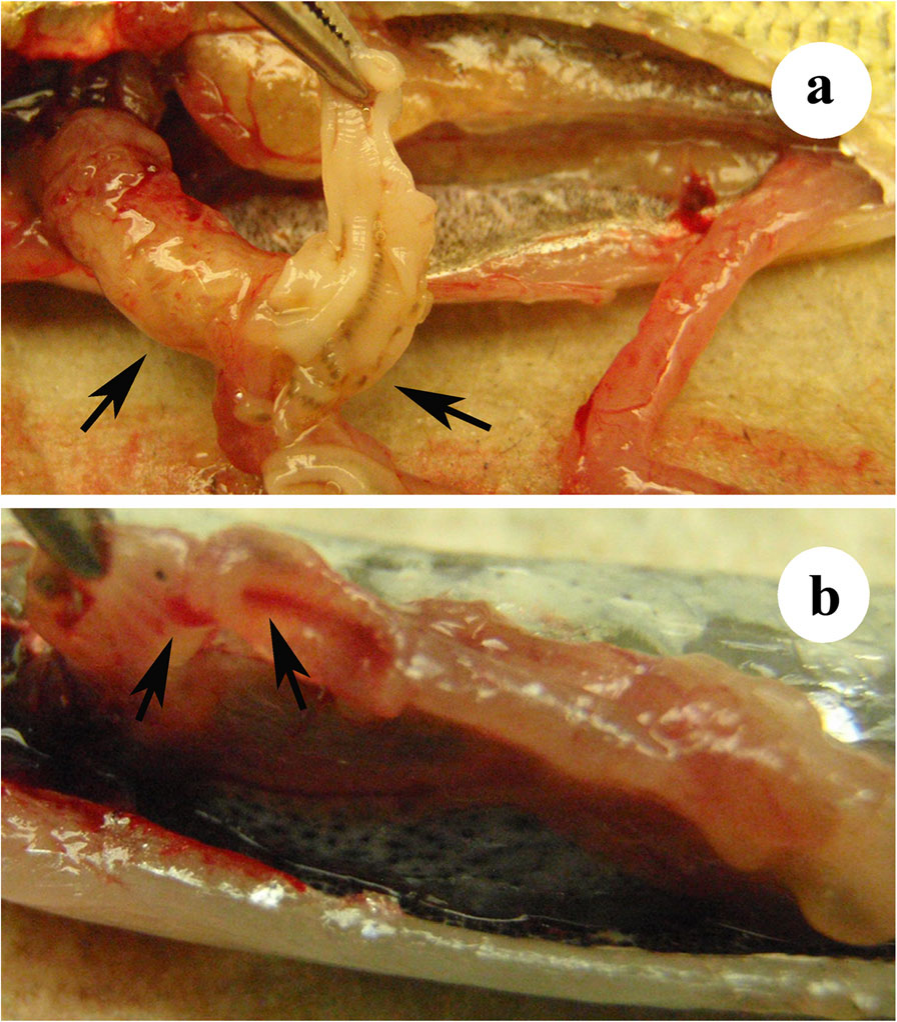
*Schyzocotyle acheilognathi* in the intestine of baitfish. **a** Golden shiner (*Notemigonus crysoleucas*) exhibiting distended, transparent and occluded intestine (arrows). **b** Emerald shiner (*Notropis atherinoides*) exhibiting transparent intestine with hemorrhage (arrows)

The anterior portion of intestine was the site where *S. acheilognathi* was attached to the intestinal walls, whereas their bodies extended throughout the entire length of the intestine. Infected fish exhibited enlarged, distended abdomens, whereby worms were frequently visible with the naked eye through the transparent alimentary canal wall (Fig. 2a). Some infected fish also showed hemorrhages within the intestinal wall (Fig. 2b).

**Fig. 3:**
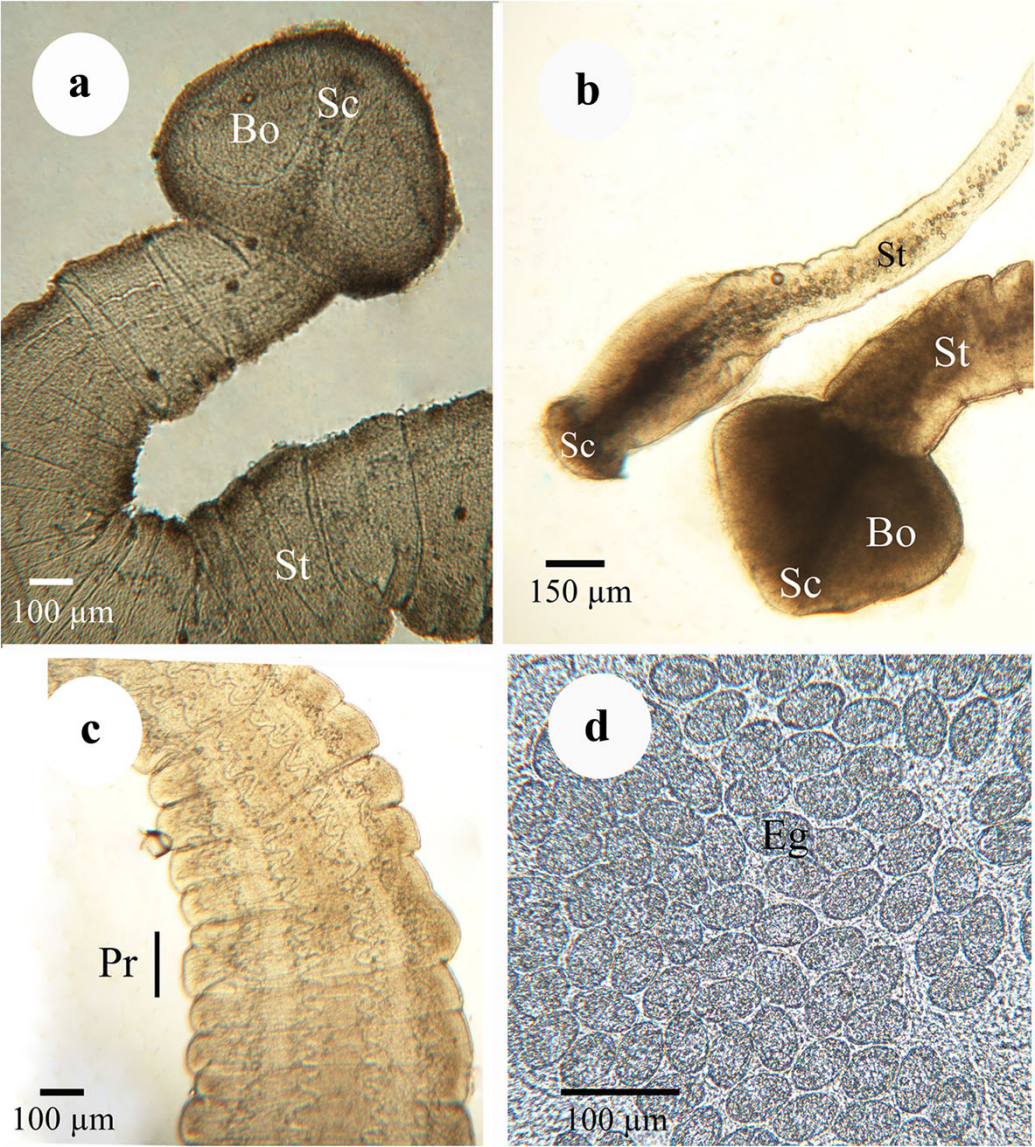
Light microscope micrographs of the Asian fish tapeworm *Schyzocotyle acheilognathi*. **a** Mature *S. acheilognathi*: note pyramidal-shaped scolex (Sc), bothria (Bo) and strobila (St). **b** Mature and immature *S. acheilognathi* found concomitantly in emerald shiner (*Notropis atherinoides*; identity of both worm stages was confirmed by gene sequence analysis). **c** Mature segmented strobila (St) with proglottids (Pr) of *S. acheilognathi* found in emerald shiner. **d** Eggs (Eg) within proglottid of *S. acheilognathi* found in golden shiner (*Notemigonus crysoleucas*)

Both immature and mature *S. acheilognathi* were recovered from the intestine of emerald, golden and sand shiners. The mature *S. acheilognathi* were identified based upon the presence of a scolex lacking suckers but with two bothria (Fig. 3a, b), the absence of a distinct neck, segmentation of the strobila (Fig. 3c), and the presence of a scolex that was wider than the anterior portion of the strobila (Fig. 3a, b). Frequently, the gravid proglottids at the distal portion of the strobila were filled with eggs (Fig. 3d). Immature *S. acheilognathi* individually varied in their development ranging from worms with a poorly developed scolex and a non-segmented body (Fig. 3b), to worms with a well-developed scolex with their segments containing non-developed reproductive organs. The majority of *S. acheilognathi* found in this study were immature. Gravid worms were found in 16 emerald shiners from 9 out of 40 infected lots purchased from November 2015 to February 2016 and in 14 golden shiners from 7 infected lots purchased during September to November 2015 and May 2016.

**Fig. 4:**
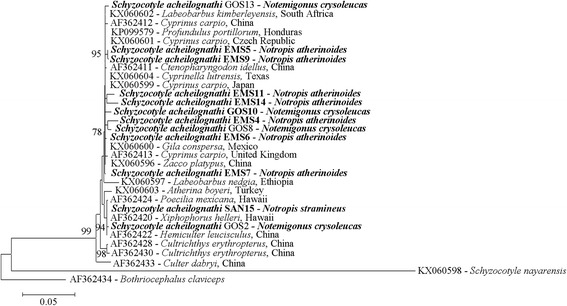
Dendrogram depicting the relationships of twelve cestodes. Cestodes recovered from emerald shiner (*Notropis atherinoides*), golden shiner (*Notemigonus crysoleucas*) and sand shiner (*Notropis stramineus*) that were collected from baitfish in Michigan were compared to 20 reference sequences (18 *Schyzocotyle acheilognathi* reference sequences, 1 *Schyzocotyle nayarensis* sequence, and 1 *Bothricephalus claviceps* sequence as an outgroup). The internal transcribed spacer region (ITS1-5.8S-ITS2) with approximate size of 1.4 kb was used as a molecular marker. The dendrogram was generated in MEGA6 [24] using neighbor-joining [25], whereby evolutionary distances were assessed via the Tamura-Nei method [37] with gamma distribution as determined using the lowest Bayesian information criterion value. The final data set contained a total of 1124 positions (complete deletion option) and only bootstrap values ≥ 70 (1000 replicates) are displayed at the nodes. Each sequence displayed the accession number, host species and origin *Scale-bar:* number of substitutions per nucleotide site

A near complete stretch of the ITS1-5.8S-ITS2 region for 12 representative specimens retrieved from the three fish species in the course of this study confirmed their identity of *S. acheilognathi*. As depicted in Fig. 4, the baitfish *S. acheilognathi* sequences fell within the well-supported clade comprised of *S. acheilognathi* reference sequences that were recovered from multiple geographical locations (e.g. South Africa; Texas, USA; Hawaii; Mexico; the Czech Republic; China; Honduras; Japan; the United Kingdom; Ethiopia; and Turkey). However, within *S. acheilognathi* clade, some genetic heterogeneity was noted, albeit slight as evidenced by the relatively small genetic distances between the sequences (Fig. 4).

### Presence of *S. acheilognathi* in Michigan retail baitfish shops

*Schyzocotyle acheilognathi* was regularly detected in baitfish collected from bait shops throughout Michigan (Fig. 1). Specifically, 48 of 90 (53.3%) lots examined resulted in the presence of *S. acheilognathi* (Table 1), with the majority of the positive samples (58.2%) originating from retail shops in the Lower Peninsula and a lower percentage (18.2%) of positive samples collected from baitfish shops in the Upper Peninsula.

**Fig. 5:**
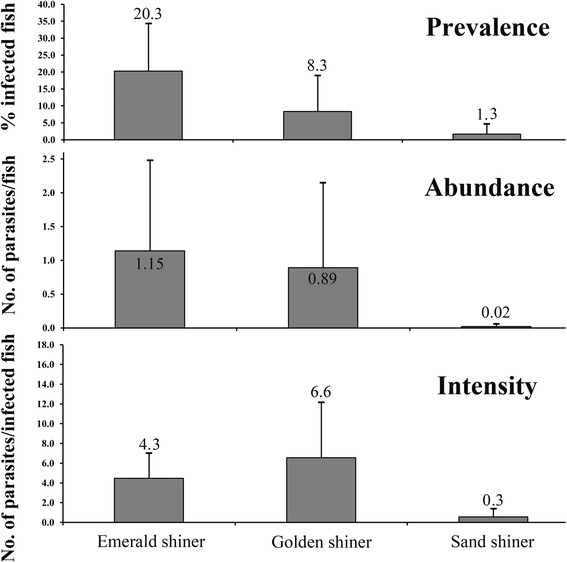
Mean prevalence, mean abundance and mean intensity of *Schyzocotyle acheilognathi* infection in three baitfish species. Baitfish species emerald shiner (*Notropis atherinoides*), golden shiner (*Notemigonus crysoleucas*) and sand shiner (*Notropis stramineus*) were sampled from Michigan baitfish retail vendors

The prevalence of *S. acheilognathi* was variable throughout the state (Fig. 1) and significantly differed among the positive baitfish species collected (*F*_(2,54)_ = 6.6, *P* = 0.003). The mean within lot prevalence was highest for the emerald shiners (20.3 ± 14.0%; Table 1; Fig. 5), which was significantly greater than that for the golden shiners (*P* = 0.02) and the sand shiners (*P* = 0.02), although the sample size was limited for the sand shiners (*n* = 3) and therefore reduced the power of the analysis. The mean within lot prevalence value for the golden shiner (8.3 ± 10.7%; Table 1; Fig. 5) was higher than for the sand shiners (1.3 ± 2.6%; Table 1; Fig. 5), but did not differ significantly (*P* = 0.62).

Intensity, similar to prevalence, differed significantly among baitfish species examined during the course of this study (*F*_(2,54)_ = 4.3, *P* = 0.019). The golden shiners had the highest mean intensity (6.6 ± 6.7; Fig. 5) which was significantly higher than the mean intensity in the sand shiners (*P* = 0.02), which had a mean of 0.3 ± 0.7. Although *S. acheilognathi* intensity was highest in the golden shiners, there was no significant difference when compared to the intensity found in the emerald shiners (*P* = 0.17). The emerald shiners had a mean intensity of 4.3 ± 2.6 (Fig. 5).

The abundance of *S. acheilognathi* did not differ significantly among the positive baitfish species collected (*F*_(2,54)_ = 1.46, *P* = 0.24). The emerald shiners had the highest mean abundance (1.15 ± 1.34; Fig. 5), but was only slightly greater than the golden shiners (0.89 ± 1.27) and the sand shiners (0.02 ± 0.05).

The three baitfish species that were infected with *S. acheilognathi* were emerald, golden and sand shiners from both in- and out-of-state sources. Specifically, the infested species from in-state sources were primarily lots of emerald shiner (*n* = 36) with some sand shiners (*n* = 3), while the out-of-state sources were predominantly lots composed of golden shiners (*n* = 28) and to a lesser extent emerald (*n* = 5) and sand (*n* = 1) shiners.

The source of where baitfish were originally collected in the wild significantly influenced the prevalence (*F*_(2,89)_ = 15.4, *P* < 0.01) and abundance (*F*_(2,89)_ = 4.6, *P* = 0.01), but not the intensity (*F*_(2,89)_ = 2.2, *P* = 0.12) of *S. acheilognathi* in the bait trade. *Schyzocotyle acheilognathi* prevalence and abundance were significantly greater for baitfish that were originally collected from in-state sources. Specifically, in-state sources (i.e. originating from MI waters; *n* = 43) had a mean within lot prevalence of 17.7% and abundance of 1.0, whereas out-of-state sources (i.e. non-MI waters; *n* = 47) had a mean within lot prevalence of only 3.5% and an abundance of 0.3. Two lots obtained were of unknown collection origin as the bait shops permanently closed before we conducted our follow-up communications to determine source.

## Discussion

After invading North America in 1975 [10], it took a quarter century and 600 miles of distance for *S. acheilognathi* to be detected in the Great Lakes basin, where it was found in a single fathead minnow specimen from Peter Lake near the Wisconsin-Michigan border in 2001 [11]. Since this initial report, *S. acheilognathi* has expanded to several other sites in the Great Lakes basin, where it has been found to infect emerald shiners, spottail shiners, mimic shiners [*Notropis volucellus* (Cope, 1865)], sand shiners, bluntnose minnows [*Pimephales notatus* (Rafinesque, 1820)] and common shiners [*Luxilus cornutus* (Mitchill, 1817)] [12, 16, 28]. This study is the most comprehensive study performed that inspects baitfish retail stores in North America in terms of the number of vendors, lots, and individual fish sampled and examined for the presence of *S. acheilognathi*. Our findings demonstrate that *S. acheilognathi* is present and widely distributed in cyprinid baitfish. Our findings indicate that the end of the trade custody chain (i.e. baitfish retail shops) represents a source for the potential introduction of *S. acheilognathi* via angler-purchased and potentially released baitfish.

Considering the ease with which *S. acheilognathi* can be disseminated due to its wide range of intermediate and definitive hosts, one would expect that *S. acheilognathi* will continue its expansion, not only in the Great Lakes, but also in inland lakes and waterways of Michigan. The potential increased risk in *S. acheilognathi* spread via the baitfish trade is indeed alarming, particularly because the pathological consequences of this worm can lead to intestinal inflammation, blockage, perforation, lack of absorption, and ultimately death [29–33]. Additionally, since 61 of the 159 fish species residing in the Great Lakes basin are threatened or endangered [34], an explosive expansion of a generalist parasite such as *S. acheilognathi* through the baitfish trade could exacerbate the tenuous status of the fish stocks at risk. In the same context, many important fish stock enhancement programs in the Great Lakes are fed with purchased baitfish from private sources in state and federal hatcheries prior to their stocking in public waters. This practice may expose these fish to a potentially lethal, debilitating parasite infections, and through widespread stocking of these fish, lead to further expansion into new geographic areas.

In this study, *S. acheilognathi* was detected in only three of eight cyprinids: emerald, golden and sand shiners. This finding, however, is, not a measurement of differential species susceptibility. For example, *S. acheilognathi* was found in fathead minnows by Choudhury et al. [11] and in spottail shiners by Muzzall et al. [12], whereas these two fish species were free of *S. acheilognathi* in our study. Emerald shiners had the highest prevalence of *S. acheilognathi* in our study, which coincides with the findings of Muzzall et al. [12] in wild emerald shiners collected from the southeastern region of Michigan. Macrogliese et al. [16] also observed *S. acheilognathi* prevalence to be the highest in emerald shiners collected from the wild and from six retail baitfish stores. Notably, the sum of these studies points to the emerald shiner as a suitable host to target in future *S. acheilognathi* surveillance studies. Likewise, the abundance of the golden shiner throughout the Great Lakes region, with wide distribution in both inland waters and the Great Lakes [35], makes this species suitable for monitoring the prevalence and spread of this invasive species in the Great Lakes basin. Golden shiners are particularly important in the imported baitfish trade and as such, would be important in conducting surveillance for *S. acheilognathi* in imported baitfish. The sand shiner could also be a good surveillance species to target based on a prevalence of 28% reported by Muzzall et al. [12].

## Conclusions

We documented that the baitfish trade may be contributing to the expansion of *S. acheilognathi* in the Great Lakes basin. The high prevalence of *S. acheilognathi* in the retail bait shops combined with the high frequency of baitfish use among anglers results in a high risk for new introductions [4]. This risk is particularly elevated when considering that anglers have been documented to release unused baitfish at rates as high as 41% [36]. As such, there is a need for managerial intervention to abrogate or slow down the rate of this pathogen expansion. In Michigan, all baitfish imported from out-of-state or harvested from Michigan waters require a health certification for each pathogen concern, especially VHSv. The inclusion of *S. acheilognathi* as a pathogen of concern for baitfish health certification should be considered. Moreover, to reduce the risk of spreading this pathogen, baitfish should only be collected from sites continuously monitored to be free from *S. acheilognathi.* In addition to best management practices during collections, it is critical to enforce existing regulations that prohibit the dumping of unused baitfish back into public waters. Public educational campaigns should also complement enforcement actions to decrease the unused baitfish release behavior that is common among anglers. Based on our findings, we recommend a combination of increased surveillance, implementation of best management practices, additional enforcement, and an increased outreach campaign to ensure that *S. acheilognathi* does not spread further throughout the Great Lakes region via the baitfish trade.
